# Supplementary material for: Liquid Metal-Based Flexible and Wearable Sensor for Functional Human–Machine Interface
Source: Micromachines (Basel). 2022 Aug 29;13(9):1429. doi: 10.3390/mi13091429 (PMC9504365; doi:10.3390/mi13091429)
Supplement: Supplementary file 1 [file micromachines-13-01429-s001.zip › 2.Supplementary Information.pdf]

Supplementary Information

Liquid Metal-Based Flexible and Wearable Sensor for Functional Human – Machine Interface

Ye Tao[a], Feiyang Han[a], Changrui Shi[a], Ruizhe Yang[a], Yixing Chen[a] and Yukun Ren[a,b]

[a]School of Mechatronics Engineering, Harbin Institute of Technology, West Da-zhi Street 92, Harbin 150001, People's Republic of China.

[b]State Key Laboratory of Robotics and System, Harbin Institute of Technology, West Da-zhi Street 92, Harbin, 150001, People's Republic of China.

This material gives supporting information in the paper:

SI Appendix 1. Design and fabrication information of the flexible sensor.

SI Appendix 2. Introduction of equipment used in sensor performance test experiments.

SI Appendix 3. Theoretical research and simulation analysis of flexible sensors.

SI Appendix 4. Flexural fatigue test of the flexible sensor.

SI Appendix 5. Composition and control principle of Bluetooth car.

SI Appendix 6. Principle of resistance measurement.

SI Appendix 7. Sensor arrangement on glove joint and its corresponding control function.

Supplementary movie:

Movie S1. Demonstrate liquid metal injection into microchannels.

Movie S2. Fatigue test for bending performance of the sensor.

Movie S3. The Demonstration of wireless sensor gloves controlling car movement.

SI Appendix 1. Design and fabrication information of the flexible sensor

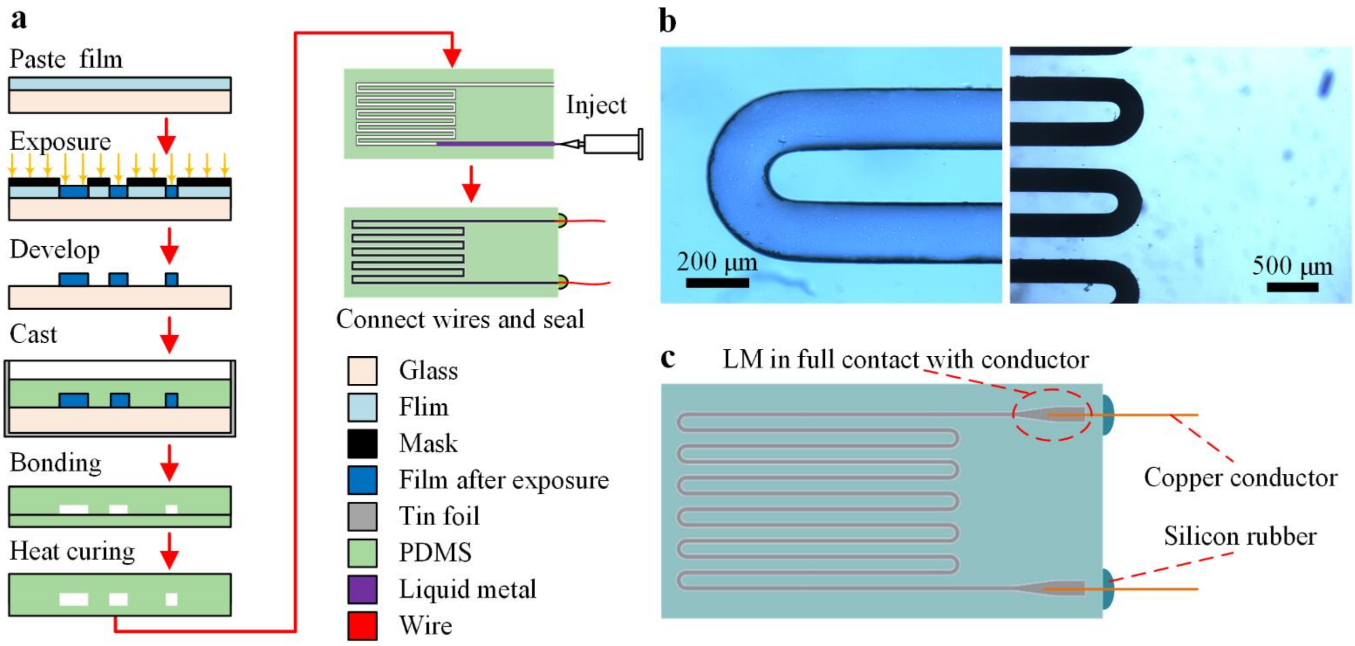

Figure.S1 According to the use ratio of the matrix: curing agent: silicone oil =20:1:2, configure PDMS and make a flexible sensor process. (a) Process of making flexible sensors by a soft lithography method. (b) The sensor channel at 10x magnification, the left is the channel where the liquid metal has not been injected, and the right is the channel after the liquid metal has been injected. (c) The microchannel of the sensor is connected with copper wires.

The specific production process of the sensor is as follows:

(1) Photoengraving mask making: Draw patterns in the software according to the shape of designed microchannels, and process the patterns into photoengraving masks.

(2) Stick Dupont membrane: Turn off all white light sources and turn on sodium for yellow light. Drop a few drops of deionized water on the surface of the smooth float glass. Paste the prepared DuPont film on the surface of the float glass. The thickness of the DuPont film is: h =38 μm and the number of layers is n, then the height of the channel is: $H = n \cdot h$. Each layer of mold should be compacted more than 2 times by a plastic sealing machine to ensure that moisture and gas under Dupont film can be removed to achieve tight sticking.

(3) Lithography: Place the mask on the surface of the Dupont film, cover it with a piece of glass and secure it. Turn on the metal halide guide lamp, and after the light source is stable for about 100 seconds, put the DuPont film under it for exposure. Each DuPont film needs 6 seconds to realize exposure, and the total exposure time is t=6n.

(4) Development: Place the DuPont film in a solution of Na_2CO_3 (1-3 wt%) and wash the channel. Na_2CO_3 will dissolve the unexposed DuPont film to form the mold of the microchannel. A single development was completed in 5 minutes. The developed channel mold is observed under a microscope. When the shape of the channel is intact and there is no obvious pollution, the development is successful. Finally, the channel mold can be used by heating it at 80°C for 5 min and drying it in the air-blast drying oven.

(5) PDMS pouring: The glass sheet with channel mold is wrapped in aluminum foil paper with an

upper side opening. After cleaning the surface of the channel die, place the die in a vacuum kettle. Use a pipette gun to pour 20-50 μL dimethyldichlorosilane into the vacuum kettle and vacuum for 3 minutes. Then restore the chamber pressure for natural deposition for 7 minutes. Mix the PDMS matrix and curing agent evenly according to the required proportion, weigh on the electronic scale, and pour into a certain quality of PDMS. Vacuum treat the PDMS for 20~30 minutes to ensure that the PDMS has no bubbles and the surface is horizontal. Put it into a blast drying oven and heat it at 80 °C for 2 hours to cure. After complete curing, use a knife to cut the PDMS and take out the PDMS with the copied channel.

(6) Bonding: Pack the float glass in aluminum foil paper in the same way, pour an appropriate amount of PDMS, remove bubbles, and heat it in a blast drying oven for 5-7 minutes for semi-curing. Fit the PDMS with the channel on the upper layer with the PDMS on the semi-cured bottom layer, and then heat it in an air blast drying oven at 80 °C for 2 hours to ensure complete adhesion and curing. After taking out and cooling the PDMS, cut out the part that duplicates the microchannel. The criteria for successful bonding of two-layer PDMS are: (a) there are no bubbles in the channel part of the bonding interface; (b) There is no PDMS blockage in the channel; (c) Two layers of PDMS will not be easily stripped; (d) The sensor will not be damaged under large deformation.

(7) Liquid metal injection: Take syringes with appropriate specifications and two syringe needles. A needle is inserted into the PDMS, and one end of the channel forms an injection outlet. The syringe absorbs clean eutectic gallium indium alloy (EGaIn), and the other end of the channel is used as the inlet. Then, liquid metal is injected, and an additional appropriate liquid metal is injected to increase the pressure inside the channel, to improve the anti-interference ability of the sensor and the response speed to stimulation.

(8) Connecting the wire and sealing: Insert the wire into the channel along the injection port of the needle, ensure that the wire is in full contact with the LM storage area, fully apply silicone rubber special adhesive at the wire connection, and let it stand for 2 hours until the adhesive solidifies to realize sealing.

SI Appendix 2. Introduction of equipment used in sensor performance test experiment

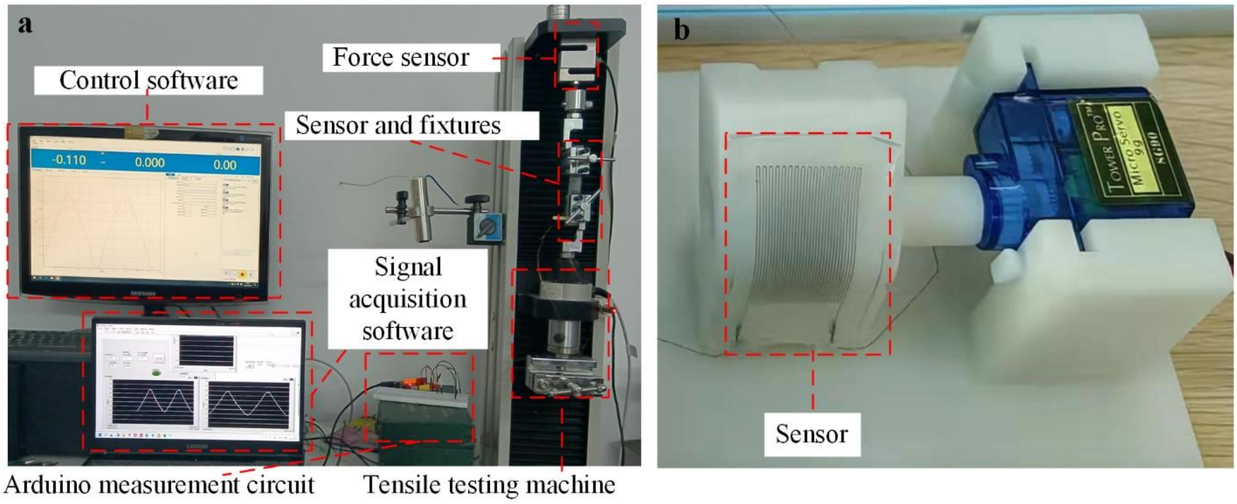

Figure. S2 The device on the left is used to test the elastic modulus of PDMS, and measure the tensile properties of linear sensors and the compression properties of helical sensors. The device on the right is used to test the bending and fatigue properties of linear sensors. (a) Electronic universal testing machine measuring platform. (b) Physical drawing of the bending device.

The electronic universal testing machine can load the sensor and can realize the displacement, deformation, and force control of the loading end through special software, combined with the acquisition system, sensor resistance, deformation, load, and other data collection (Figure S2(a)).

The composition of the bending mechanism is shown in Figure 3(a). The steering gear is the power source of the device, and its output end is assembled with the rotating shaft, to output power to the upper swing head. The swing between the upper and lower swing heads simulates the bending action of the knuckle, and the knuckle joint is simplified as a hinge, and the bending test is conducted on the sensor attached to it. The physical object is shown in Figure S2(b).

SI Appendix 3. Theoretical research and simulation analysis of flexible sensor

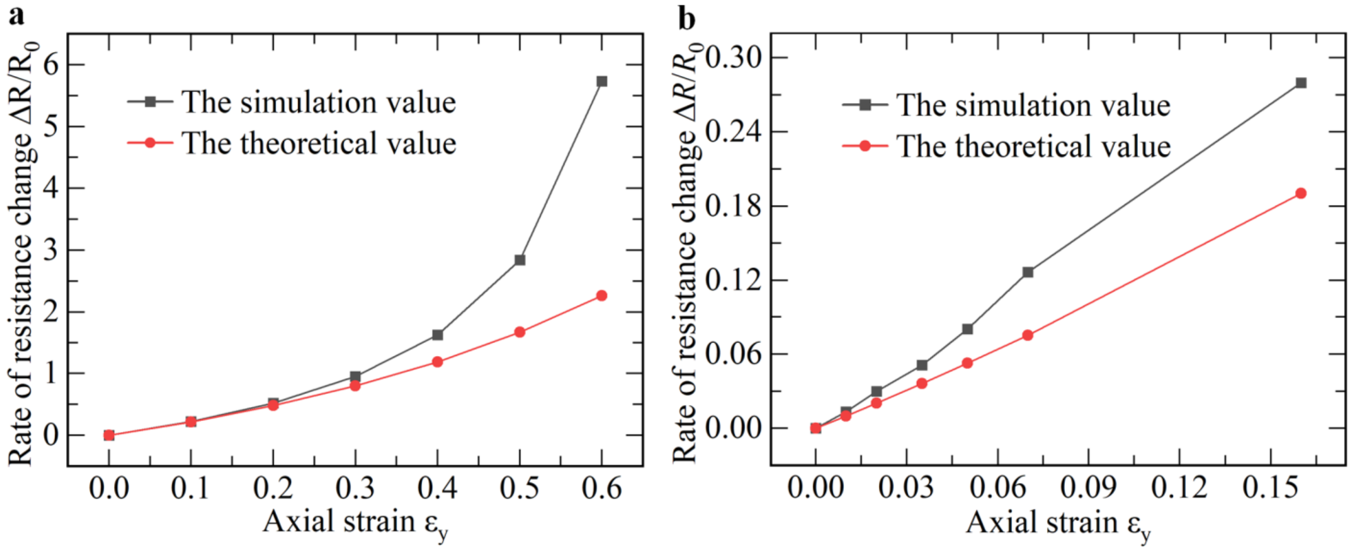

Figure. S3 Firstly, the theoretical resistance of the flexible sensor is analyzed. Then theoretically analyze the relationship between the resistance signal and the load when the sensor is stretched and compressed. Finally, a simulation model is established, and through numerical simulation analysis, the differences between the simulation results and theoretical analysis are compared. (a) Theoretical and simulation strain-stress curves for linear sensor stretching. (b) Theoretical and simulation strain-stress curves of a spiral sensor during stretching.

The cross-section size of the channel is micron level, which is far smaller than the overall size of the sensor. Therefore, we assume that the influence of the presence of liquid metal on the deformation law of the flexible body is negligible during the loading deformation process. In other words, in the process of theoretical analysis, the deformation of the flexible material still maintains the deformation law of PDMS material, and the deformation effect of the liquid metal channel is consistent with that of the flexible body of the sensor. When the sensor is stretched, the channel length and cross-sectional area are calculated according to eqn S1 and eqn S2 respectively. The deformation relation of the material is shown in eqn S3. The above formula is substituted into eqn 5 to calculate the theoretical resistance and eqn S4 is obtained.

$$L = L_0 + \Delta L = L_0(1 + \varepsilon_y) \tag{S1}$$

$$S = b \cdot H = (b_0 + \Delta b) \cdot (H_0 + \Delta H) = b_0 H_0 (1 + \varepsilon_x)^2 \tag{S2}$$

$$\varepsilon_x = -\nu \cdot \varepsilon_y \tag{S3}$$

$$R = \rho \frac{L}{S} = \rho \frac{L_0(1+\varepsilon_y)}{b_0 \cdot H_0 (1-\nu \cdot \varepsilon_y)^2} = R_0 \frac{(1+\varepsilon_y)}{(1-\nu \cdot \varepsilon_y)^2} \tag{S4}$$

where L denotes the real-time length of the sensor channel, L_0 denotes the initial length of the sensor channel, ΔL denotes the length change of the sensor channel after loading, ε_y denotes the longitudinal strain of the sensor. b denotes the channel width, Δb denotes the change of sensor channel width after loading, H denotes the channel height, ΔH denotes the change of sensor channel height after loading, ε_x denotes the radial strain of the sensor, v denotes the Poisson's ratio of the material, for PDMS materials, 0.5 is generally taken.

We use a spiral sensor to measure the surface normal pressure F, and what needs to be studied is the relationship between its normal compression and sensor resistance. Similarly, eqn 5 is used to calculate its

resistance value. When the sensor body is compressed, its thickness decreases, and its radial size increases, which leads to the increase in channel width B and length L in the section size of the channel. The strain of longitudinal compression is ε_y, and the increase of radial size is calculated as $\varepsilon_x = -\nu \cdot \varepsilon_y$, according to eqn S3. The length of the helix is positively correlated with its radial size, so it can be considered that the increase rate of helix channel length is also $\varepsilon_{x,}$ and the increase rate of channel width is also ε_x. According to the above analysis, we get that the length L, width B, and height h of the channel are calculated as follows:

$$L = L_0 \cdot (1 + \nu \cdot \varepsilon_y) \tag{S5}$$

$$b = b_0 \cdot (1 + v \cdot \varepsilon_y) \tag{S6}$$

$$H = H_0 \cdot (1 - \varepsilon_y) \tag{S7}$$

By substituting the above formula into eqn (5), the resistance R of the spiral sensor can be calculated as:

$$R = \rho \frac{L}{bH} = \rho \frac{L_0}{b_0 H_0 (1-\varepsilon_y)} = R_0 \frac{1}{1-\varepsilon_y} \tag{S8}$$

Two physical fields are required to simulate flexible sensors in simulation software: solid mechanics and current field. The deformation of flexible materials under tensile or compressive loads belongs to the category of solid mechanics, and the current module is needed to calculate the resistance of materials in simulation software. Figure S3(a) is drawn based on simulation data and eqn S4. It can be seen from the figure that when the axial strain ε_y<0.3, the simulation value and the theoretical curve can coincide. When the axial strain ε_y>0.4, the deviation begins to appear, and at the endpoint ε_y=0.6, the simulation value is more than twice the theoretical value. Figure S3(b) is drawn based on simulation data and eqn S8. As can be seen from the figure, the various rules of simulation results and theoretical analysis results are the same. However, there is a gap in the numerical value, and the simulation result is about 1.3~1.7 times the theoretical value.

SI Appendix 4. Flexural fatigue test of flexible sensor

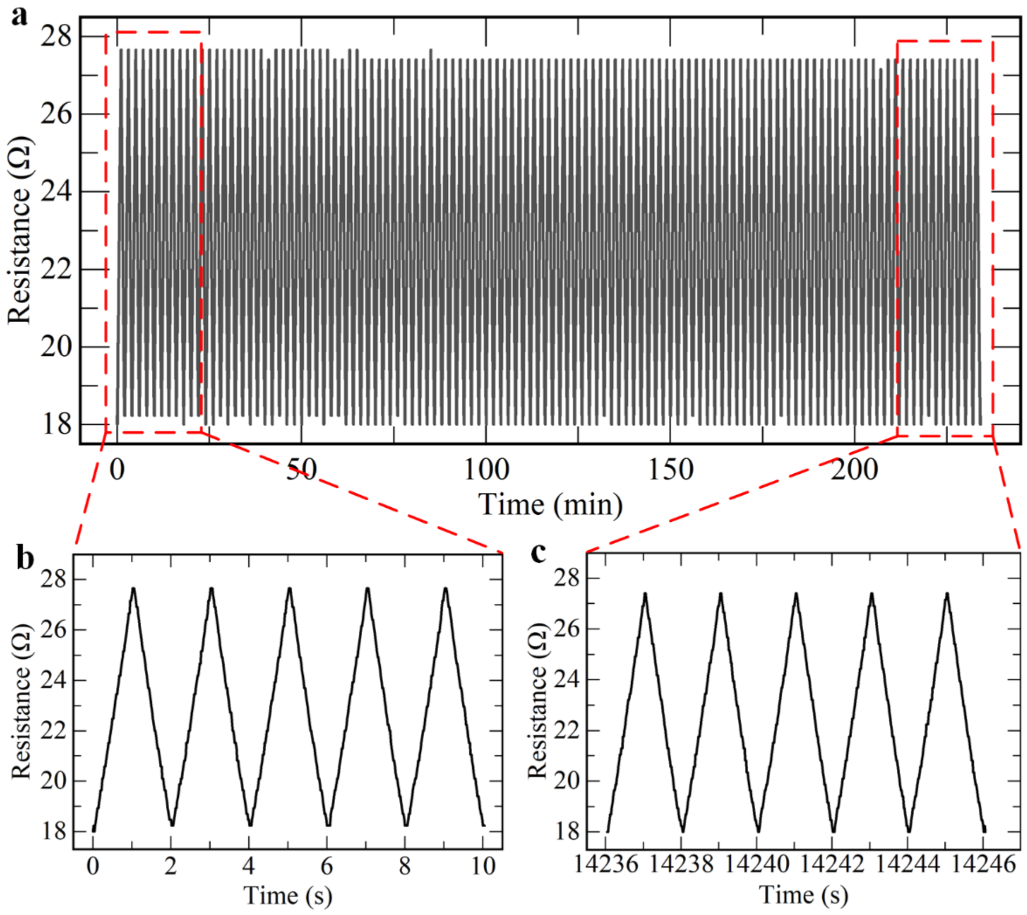

Figure. S4 To measure the fatigue performance of the sensor during bending, a bending mechanism was used to repeatedly bend the linear sensor for 4 hours. (a) Fatigue test experiment of sensor bending. (b) The initial phase of testing. (c) The end of the test phase.

Set the bending diameter as 9mm, the motion range as 0~90°, and the cycle as 2 seconds. Figure S4(a) shows the sensor signal variation in the bending process, and the local data at the beginning and end are shown in Figure S4(b) and (c).

In Figure S4(a), the resistance of the sensor remained between 18~28 Ω during the 4-hour bending process, and the range of changes was almost the same. Therefore, the sensor maintained excellent repeatability during the 4-hour continuous use. There is no fatigue or crack before and after the fatigue test, and no change in the performance of the sensor, indicating excellent fatigue resistance.

SI Appendix 5. Composition and control principle of Bluetooth car

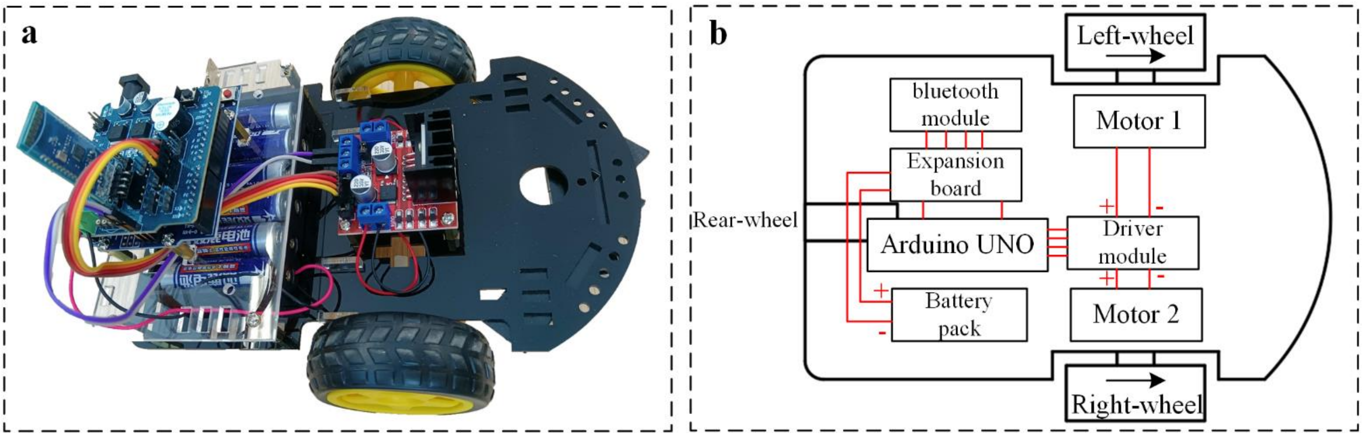

Figure. S5 The composition and control principle of Bluetooth car, the design of signal acquisition circuit, and data receiving program. (a) Physical demonstration of Bluetooth car (b) The structure of Bluetooth car

The Bluetooth car used is shown in Figure S5(a), and its composition principle is shown in Figure S5(b). The working principle is as follows: the computer sends the signal to the Arduino development board through the serial port via Bluetooth. The four ports of the Arduino are set as the output ports of the pulse signal. The drive module receives the PWM pulse signal and outputs the corresponding voltage to control the speed of the left and right tires. When the speed of the two motors is the same, the car moves forward and backward; When the speed of the two motors is different, the car realizes the steering function depending on the differential principle. Table S1 is the main components used in building the Bluetooth car.

Table S1 Composition of the Bluetooth car

Components	Model
SCM	Arduino UNO
Bluetooth module	JDY-31
Motor drive module	L298N
Motor	130-Motor

SI Appendix 6. Principle of resistance measurement and design of signal acquisition circuit

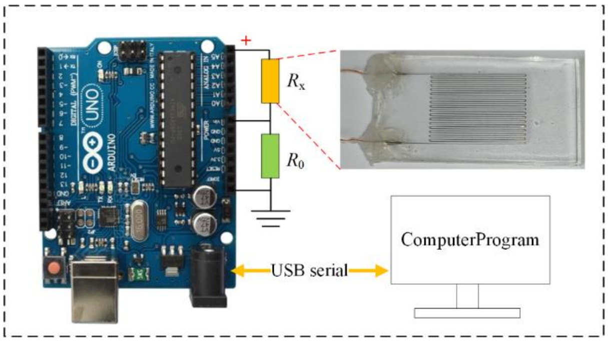

Figure S6 Principle of resistance measurement.

The circuit control system uses a Bluetooth serial port to communicate. All the hardware circuits are integrated into the gloves, and the PCB board is designed. Its size is 32×32 mm, which can be configured on the back of the hand. Figure.4 (c) shows the preview of the PCB. Connect the wires of each sensor to the FPC connector, and then connect the FPC connector to the PCB, and then insert the Bluetooth module to work.

The output current I_0 of the DC source greatly affects the acquisition accuracy of the single chip microcomputer. The A/D conversion bit of the STM32 single chip microcomputer is 12 bits, and the voltage range of the signal input point is 0-3.3V, that is, the digital signal that is converted from 0-3.3V to 0-4095 is sent through the serial port. Assuming that a total of nine linear channel sensors are used, their average initial resistance is 25 Ω. After loading deformation, their resistance R increases by 100%. Adding the comparison resistance R_0=20 Ω with a fixed resistance value, the total resistance value varies from 245 to 470 Ω. When we adjust the current output of the DC source so that the total resistance is 500 Ω, the input voltage of the last port A_9 reaches the maximum port input voltage of 3.3V, and the maximum resolution of the resistance is 500/4096 Ω =0.1221 Ω. The resolution is 0.1221·(90-0)/(50-25)=0.440 Ω/° when the sensor resistance 25~50 Ω signal changes due to a 0~90° angle bending. To achieve this precision, we need at least a current output from the DC source of I_0 = 3.3/500 = 6.6 mA. The DC power supply regulates current by shunt resistance at Vout, so the design goal of the circuit is to ensure that the DC source can output more than 6 mA of DC.

The digital signal D_n of each port is obtained after the data is segmented and converted by the software program. The digital signal is converted into port potential V_n through eqn S9.

$$V_n = \frac{3.3}{4095} \cdot D_n \tag{S9}$$

where D_n denotes the digital signal of the signal input port of the MCU.

SI Appendix 7. Sensor arrangement on glove joint and its corresponding control function

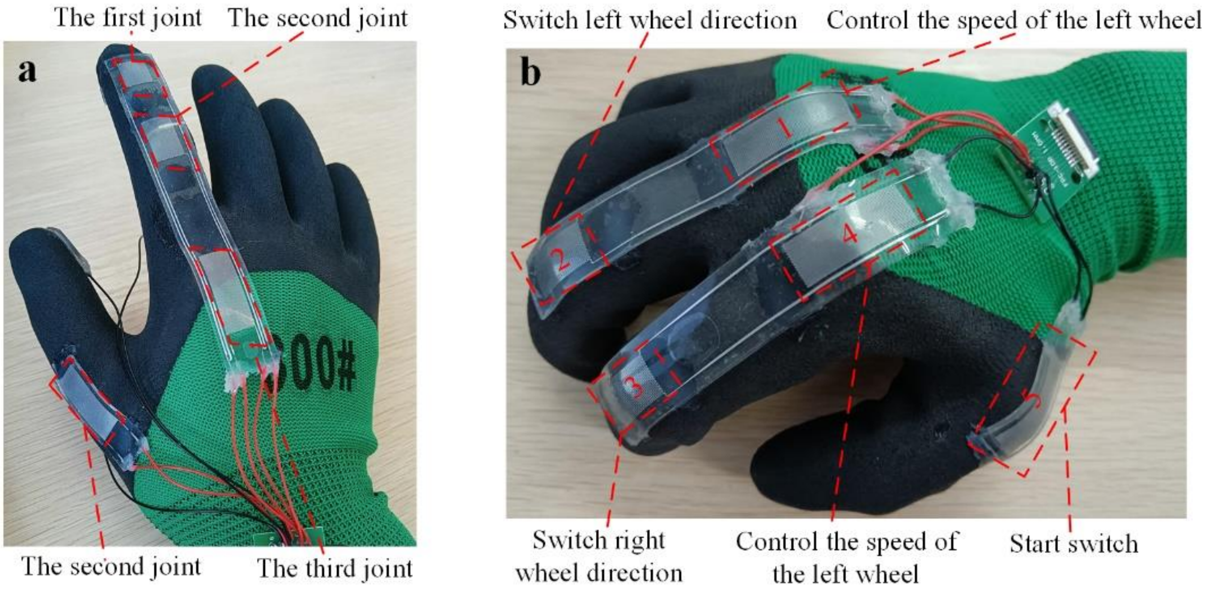

Figure. S7 This figure introduces how to control the car by finger bending to achieve different motions and speeds. (a) The placement of sensors on the thumb and index finger joints. (b) Corresponding control functions of sensors on each joint.

To make the gloves fully fit the hand and transmit the deformation to the sensor, the gloves should not be too soft, otherwise, the stress of the sensor deformation will drive the glove deformation, and the deformation can not be accurately transmitted to the sensor. The common protective gloves on the market are thick enough to carry flexible sensors and are used in experiments. The sensor is glued to the glove to form the sensor glove as shown in Figure S7. The sensor gloves contain three sensors. The sensor on the thumb is a single joint sensor. The sensors on the index and middle fingers are double-joint sensors, both are used to detect the bending angles of the second and third joints. As Figure S7(a) shows.

The setting operation mode is: the sensor on the middle finger controls the speed and positive and negative direction of the car's left wheel respectively; A sensor on the index finger controls the speed and direction of the right wheel; The single sensor on the thumb acts as the start switch of the car. When its bending angle exceeds 70°, it switches between on and off. As can be seen from the conclusion in the previous section, there is an error of -5~5° in joint bending Angle detection of gloves, so it is impossible to achieve infinitely variable speed, so graded variable speed is adopted. The corresponding relationship between the finger joint bending angle θ detected by the sensor and the tire speed V is divided into five levels. The tire speed is controlled by controlling the duty cycle of pulse PWM. Table S5 shows the relationship between tire speed levels and joint bending angles.

Table S2 Elastic modulus of PDMS under different proportion of curing agent

PDMS matrix: curing agent (wt)	20:1	18:1	16:1	14:1	12:1	10:1
Young's modulus of tension（MPa）	0.540	0.569	0.682	0.866	1.236	2.092
Compressed Young's modulus（MPa）	1.488	1.542	1.856	2.169	2.441	3.022

Table S3 Elastic modulus of PDMS under different silicone oil ratio

Silicone oil: PDMS	1:3	1:5	1:10	1:20	No silicone oil
Young's modulus of tension（MPa）	0.863	1.277	1.598	1.849	1.924
Compressed Young's modulus（MPa）	1.712	2.495	3.138	3.781	3.773

Table S4 Analysis of sensor bending data

D(mm)	ΔH_{max} (Ω)	k(Ω/°)	r_L(%)	r_H(%)	Linear fitting R-squared	Quadratic fitting R-squared
9	0.47	0.13576	1.909	3.447	0.99853	0.99947
12	0.95	0.16246	3.652	2.937	0.99781	0.99847
20	0.9672	0.28244	3.109	2.829	0.9934	0.998

Table S5 The relation between speed level and bending angle

Speed level	0	1	2	3	4	5
Bending angle (°)	[-9,9)	[9,27)	[27,45)	[45,63)	[63,81)	[81,99)
